# Multiscale Interface Effect on Homogeneous Dielectric Structure of ZrO_2_/Teflon Nanocomposite for Electrowetting Application

**DOI:** 10.3390/polym10101119

**Published:** 2018-10-09

**Authors:** Jiaxin Hou, Yancong Feng, Jinglun Liao, Wenwen Ding, Lingling Shui, Hao Li, Yao Wang, Biao Tang, Ahmad Umar, Guofu Zhou

**Affiliations:** 1 Guangdong Provincial Key Laboratory of Optical Information Materials and Technology & Institute of Electronic Paper Displays, South China Academy of Advanced Optoelectronics, South China Normal University, Guangzhou 510006, China; houjiaxin1993@outlook.com (J.H.); fengyancong@m.scnu.edu.cn (Y.F.); liaojinglun@hotmail.com (J.L.); phoenix_wenwen@163.com (W.D.); shuill@m.scnu.edu.cn (L.S.); wangyao@m.scnu.edu.cn (Y.W.); tangbiao@scnu.edu.cn (B.T.); guofu.zhou@m.scnu.edu.cn (G.Z.); 2 National Center for International Research on Green Optoelectronics, South China Normal University, Guangzhou 510006, China; 3 Department of Chemistry, Faculty of Science and Arts and Promising Centre for Sensors and Electronic Devices, Najran University, Najran 11001, Saudi Arabia; ahmadumar786@gmail.com; 4 Shenzhen Guohua Optoelectronics Tech. Co. Ltd., Shenzhen 518110, China; 5 Academy of Shenzhen Guohua Optoelectronics, Shenzhen 518110, China

**Keywords:** electrowetting, dielectric layer, interface effect, ZrO_2_ nanoparticles, Teflon

## Abstract

Electrowetting-on-dielectric is a preferred option in practical applications of the electrowetting phenomenon but limited by dielectric and breakdown performances of the dielectric layer. In the present work, a ceramic/polymer nanocomposite as a novel dielectric layer is developed to intensify the overall electrowetting performances by multiscale interface effect. Hereinto, surface fluoro-modified ZrO_2_ nanoparticles (mZrO_2_) are dispersed well in AF 1600 matrix to form a mZrO_2_@AF 1600 nanocomposite. The small addition of mZrO_2_ improves the dielectric constant of the nanocomposite, and the experimental value is larger than the theoretical value calculated by Maxwell–Garnett model, but fits well with the Rahaman–Khastgir model. The molecular dynamics simulations with the explicit model further verify the interfacial effect. Meanwhile, double contact angle modulation and higher breakdown field strength (*E*_b_) are obtained. For the three-layer sandwich structure, both the top and bottom AF 1600 layer decrease the surface roughness for better electrowetting reproducibility and wider wettability modulation. The Forlani–Minnaja theory related to the empirical relationship between *E*_b_ and thickness of dielectric layer fit well with the monolayer structure, but cannot be applied in multi-layer structures. A new relationship is proposed to guide the design of dielectric multi-layers with high breakdown field strength.

## 1. Introduction

Electrowetting is a microfluidic phenomenon that tunes the surface tension of the liquid droplet on the electrode to enable large and reversible contact angle variations via applied electric field [1,2]. Till now, it has grown into an efficient, versatile, and promising tool to regulate surface wettability of materials and shape of microdroplets, and been applied to electronic paper display [3], microfluidics [4,5], microlenses [6], fiber optics [7], and other electronic fields [8]. In these applications, electrowetting-on-dielectric (EWOD) is a preferred option more than direct electrowetting-on-a-conductor, by which a hydrophobic dielectric layer covers the electrode. High dielectric constant of layer material can strengthen the electrowetting effect in high electric fields. The resultant low surface energy and wettability can make surface microfluids flow expediently [9]. Thus, the dielectric layer is absolutely crucial to the overall performance of EWOD.

Nowadays, more and more inorganic or polymeric materials are used to present dielectric layers [10,11,12]. The former is good at high dielectric constant and low driving voltage, such as SiO_2_ [13], Si_3_N_4_ [14], silicon nanosphere [15], and mesoporous silica [16]. However, their natural defects in film-processing ability seriously hinder the manufacture or electrowetting devices. In particular, high polarity of metal oxides (e.g., ZnO [17,18,19]) even causes incident dielectric lose efficiency. On the contrary, the latter represented as Teflon AF [20,21], Cytop TM [22], Parylene-C [23], polydimethylsiloxane [24], polyimide [25], poly(ethylene terephthalate)/polyethylene [26], and photoresist Su-8 [27], are mainly limited by their relatively low dielectric constant, which is adverse to decreasing driving voltage and avoiding dielectric breakdown [28]. 

Novel dielectric composite layers for EWOD are further developing, consisting of a top hydrophobic polymer layer and bottom dielectric inorganic/polymeric layer [29,30,31,32]. The layers combine the advantages of both inorganic and polymeric insulating materials but ignore the huge differences in chemical and physical properties between the two materials. Once an electric field is applied, electric charges inevitably concentrate on the surface of hydrophobic layer with much lower dielectric constant than the dielectric layer, accelerating the lose efficiency. In addition, their weak interlayer compatibility will cause layer-to-layer miss-registration, interstice, and even separation.

Addition of ceramic fillers can improve the dielectric constant of the composite. In particular, ferroelectric ceramics with perovskite structure, such as BaTiO_3_ [33,34], possess intrinsic high dielectric constant up to hundreds and even thousands due to the spontaneous polarization. However, the polarization mainly concentrates upon the interior of fillers. The influence of fillers on the surface of material is still unclear. On the other hand, nanosized fillers are popular for the dielectric film with only sever micrometers; however, nanosized ferroelectric ceramic particles have relatively lower dielectric constant than bulk ceramics due to the phase transition from tetragonal to cubic [35]. For the devices of EWOD, the optical properties should be also considered, limiting the selectivity of type of filler.

**Scheme 1:**
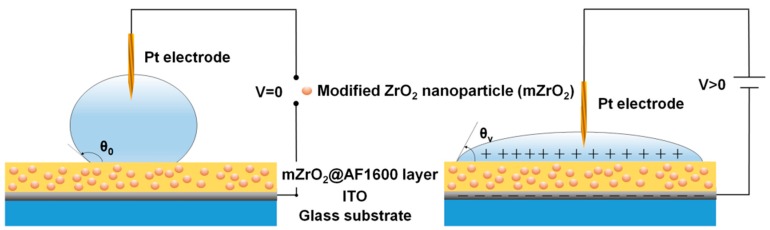
Schematic of electrowetting phenomenon of mZrO_2_/AF 1600 nanocomposite.

As a consequence, we focus on integrating both inorganic and polymeric insulating materials into one highly compatible solution, and preparing the polymer nanocomposite. Within this composite structure, surface fluoro-modified zirconia (mZrO_2_) nanoparticles with high dielectric constant [36] and high transmittance were hybridized into AF 1600 to form the dielectric nanocomposite. The electrowetting phenomenon of mZrO_2_/AF 1600 nanocomposite is presented in Scheme 1. Under the electric field, the originally hydrophobic surface changes to be hydrophilic, leading to the decrease of contact angle of droplet on the surface.

Besides the dielectric constant, the breakdown field strength also plays a key role in the performance of dielectric materials. Designing sandwich-structured ceramic/polymer nanocomposites can significantly enhance breakdown field strength [37,38]. However, the relationship between multi-layer structure and breakdown field strength keeps confused. In the present work, sandwich structure is also designed for the enhancement of breakdown field strength, and the regularity is revealed. Meanwhile, the sandwich structure also avoids ionic polarization and alters the surface property.

## 2. Experimental and Simulated Methods

### 2.1. Materials

ZrO_2_ nanoparticles (99.9%; mean size: ~20 nm) and Teflon AF 1600 were purchased from Beijing DK nano technology Co., Ltd. (Beijing, China) and DuPont Company (Wilmington, DE, USA), respectively. 1H,1H,2H,2H-perfluorooctyltriethoxysilane (97%, J&KScientific Ltd., Beijing, China) and prefluorotributylamine (84%, Beijing HWRK Chem Co., Ltd., Beijing, China) were directly used without any further purification. Benzotrifluoride (99%, Adamas Reagent Co., Ltd., Shanghai, China) was dried by vacuum distillation over calcium hydride (CaH_2_) prior to use. All other regents are of analytic grade.

### 2.2. Surface Modification of ZrO_2_ Nanoparticles

**Scheme 2:**
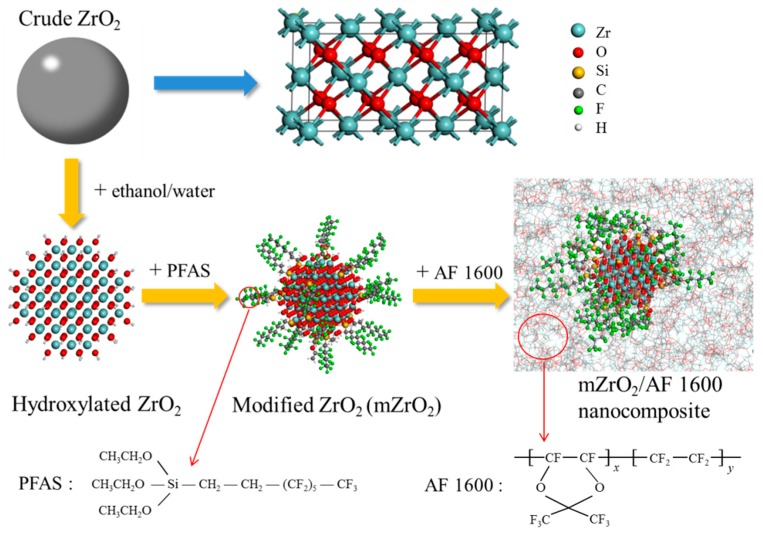
Schematic of preparation of mZrO_2_/AF 1600 nanocomposite.

ZrO_2_ nanoparticles were modified via surface coupling reaction of fluorinated silane coupling agent (see Scheme 2) as described in the literature [39]. Typically, the crude ZrO_2_ nanoparticles were rinsed three times with acetone, ethanol and deionized water in ultrasonic cleaner for 10 min, respectively, and then were dried in vacuum at 60 °C for 12 h. In dry argon atmosphere, all these nanoparticles were mixed with 1H,1H,2H,2H-perfluorooctyltriethoxysilane at 1:1.5 mass mole^−1^ ratio in benzotrifluorideby magnetic stirring at room temperature for 72 h. Finally, the mZrO_2_ product was rinsed two times with ethanol and acetone, respectively, and then were dried in vacuum at 50 °C for 24 h.

### 2.3. Characterization of ZrO_2_ Nanoparticles

Scanning electron microscope (SEM): The morphologies of crude and modified ZrO_2_ nanoparticles were observed using a ZEISS Ultra 55 (Germany) with the ions energy of 5 kV. Samples were respectively prepared by drying the ethanol solution of crude ZrO_2_ and the benzotrifluoride solution of mZrO_2_ with the same concentration of 0.1 mg mL^−1^ on the clean glass flake adhered to the copper platform.

Static contact angle measurements: Two kinds of ZrO_2_ nanoparticles were respectively tableted into plates using manual powder tablet press (769YP-15A, KEQI, Tianjin KEQI High & New Technology Corporation, Tianjing, China). Then, a 10 μL droplet of ultrapure water from a hydrophobized needle of a micro-syringe was dropped onto the surface of the plates. The resulting contact angle was recorded by a contact angle meter (JC2000C, POWEREACH, Shanghai, China), and measured by an image analysis software (DSA10Mk2 drop shape analysis system, Krüss Hamburg, Germany).

Fourier infrared spectrometer (FT-IR): The surface variation between the crude and modified ZrO_2_ nanoparticles was identified by FT-IR (Tensor 27, Bruker, Karlsruhe, Germany) in a spectral range of 4000–400 cm^−1^ with a resolution of 2 cm^−1^ using a potassium bromide plate.

### 2.4. Compatibility Test of ZrO_2_ with AF 1600

The compatibility tests were performed using differential scanning calorimetry (DSC; DSC1, METTLER TOLEDO, Zurich, Switzerland) within a temperature range of 25 to 200 °C at a heating rate of 10 °C min^−1^ under nitrogen gas (flow rate: 10 mL min^−1^).

### 2.5. Preparation on mZrO_2_@AF 1600 Layer

Typically, a determined weight of mZrO_2_ nanoparticles was uniformly dispersed in prefluorotributylamine solution of AF 1600 (3.7 wt %) by high intensity ultrasound (VCX150PB, SONICS, Newtown, PA, USA). Afterwards, the mixture was coated on the surface of indium tin oxide (ITO) glass using a spin coater (KW-4A, Institute of Microelectronics of the Chinese Academy of Sciences, Beijing, China) with a fixed rotating speed of 1200 rpm. Finally, the coated slide was dried in a vacuum at 100 °C for 2 h to remove the residual organic solvent thoroughly. Pure AF 1600 layer, double-, and three-layer structures were fabricated by repeating the same process.

### 2.6. Characterization of mZrO_2_@AF 1600 Layer

Capacitor measurements: The capacitor measurements were performed by inputting signal of alternating current (AC; 0.5 V, 1 kHz) using an impedance analyzer (6500B, WAYNE KERR, London, UK). The positive pole of the impedance analyzer was attached to the platinum (Pt) probe that was inserted into a 10 μL droplet of 0.1 M sodium chloride (NaCl) aqueous solution on the layer surface, and the negative pole was attached to the ITO side of the substrate. The real-time capacity values were displayed on the screen of the impedance analyzer until remaining constant.

Surface profile analysis: The layer thickness, surface topography and roughness were measured by three-dimensional (3D) surface profiler (DCM8, Leica, Biberach, Germany) with a 50-fold objective.

Electrowetting test: The electrowetting tests were performed using digital regulated direct current (DC) power supply (CE0400010T, Earthworm Electronics, Shanghai, China) (see Scheme 1) with the same pole connection mentioned above in the capacitor measurements. As the applied voltage increased from 0 V to the breakdown voltage (*V*_b_) value (see the following *V*_b_ measurements) with the step of 5 V, the real-time change of NaCl droplet was recorded by contact angle meter and analyzed using an image analysis software.

Dynamic contact angle measurements: Typically, a 10 μL droplet of ultrapure water from the hydrophobized needle of a micro-syringe was dropped onto the layer surface. During the movement of the objective table, the resulting advancing angle, and receding angle was recorded by contact angle meter, and measured by image analysis software.

*V*_b_ measurements: The *V*_b_ measurements were performed using a digital regulated DC power supply with the same pole connection mentioned above in the capacitor measurements. Along with increasing DC voltage, the real-time changes of NaCl droplet were observed by contact angle meter till a few tiny bubbles generated in the droplet. At this time, the applied DC voltage is viewed as *V*_b_. The final *V*_b_ is the average of at least five measured values with mean square error less than 5 V.

### 2.7. Simulated Method

Scheme 2 shows the structure of Teflon AF. For the AF 1600, the ratio of *x*/*y* is 13/7. In our models, we built the AF 1600 molecules with *x* = 13 and *y* = 7. There were 100 molecules in the pure AF 1600 system. For the mZrO_2_/AF 1600 nanocomposite, the spherical ZrO_2_ (containing 87 Zr atoms and 174 O atoms) with a radius equaling to approximately 8 nm was built, and there were 100 AF 1600 molecules. The ZrO_2_ particle was grafted by 20 PFAS molecules. The simulation was run at 500 K for 0.2 ns in the NPT ensemble to obtain sufficiently relaxed structures. Then, the system was run at 300 K in the NPT ensemble for 0.2 ns to obtain an equilibrated density. Finally, the simulation was run at 300 K in the NVT ensemble for 0.2 ns and the following 0.3 ns data were recorded for analysis.

## 3. Results and Discussion

As well-known, homogeneous nature is the prerequisite to optimize the overall performance of polymer composite, which is mainly determined by matrix compatibility. However, fluoropolymers represented as Teflon series is generally incompatible with other common materials. Therefore, “surface ligand engineering” is often introduced to improve the compatibility between filler and framework material [40,41]. Here we employed the coupling of 1H,1H,2H,2H-perfluorooctyltriethoxysilane (PFAS) to the hydroxylated surface of ZrO_2_ nanoparticles to achieve surface fluorination (see Scheme 2).

**Figure 1:**
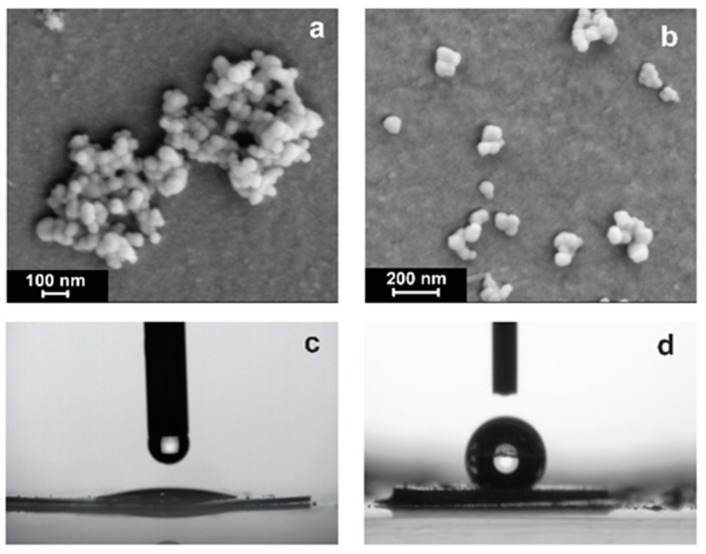
SEM and static water contact angle images of ZrO_2_ nanoparticles before (**a** and **c**) and after surface fluorination (**b** and **d**), respectively.

**Figure 2:**
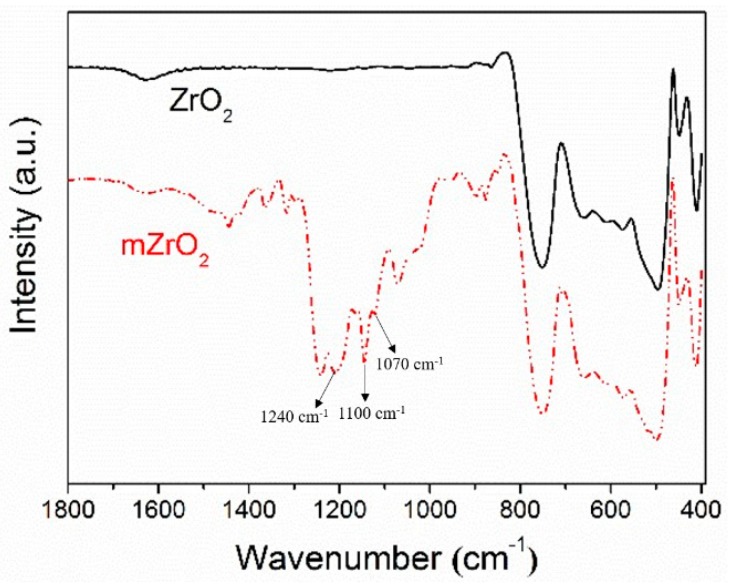
FT-IR spectra of crude zirconia (ZrO_2_) and surface fluoro-modified zirconia (mZrO_2_). The related peaks listed below: 1240–1100 cm^–1^ (νs, C–F on PFAS) and 1070 cm^–1^ (νas, Si–O linked with ZrO_2_).

In general, interfacial incompatibility exists due to the hydrophilicity of filler surface and hydrophobicity of polymer, leading to aggregation of nanoparticles [42,43]. Naturally, the introduced fluorocarbon chains [44] with a similar component as matrix chains will weaken the interaction within ZrO_2_ nanoparticles against aggregation, just like what appears in Figure 1b. Here, there is no big aggregation of fluorosilane-modified nanoparticles (mZrO_2_), opposite to what the crude ZrO_2_ ones did in Figure 1a. In particular, the static water contact angle rises from less than 10° to 135°, i.e., the superficial nature of the nanoparticles alters from hydrophilic to hydrophobic (see Figure 1c,d). It indicates that surface modification greatly changes the wettability of ZrO_2_. Moreover, the C–F stretching vibration (1240–1100 cm^−1^), and Si–O antisymmetric stretching vibration (1070 cm^−1^) in the FT-IR spectra (see Figure 2) also provide convincing evidence on surface fluorination.

**Figure 3:**
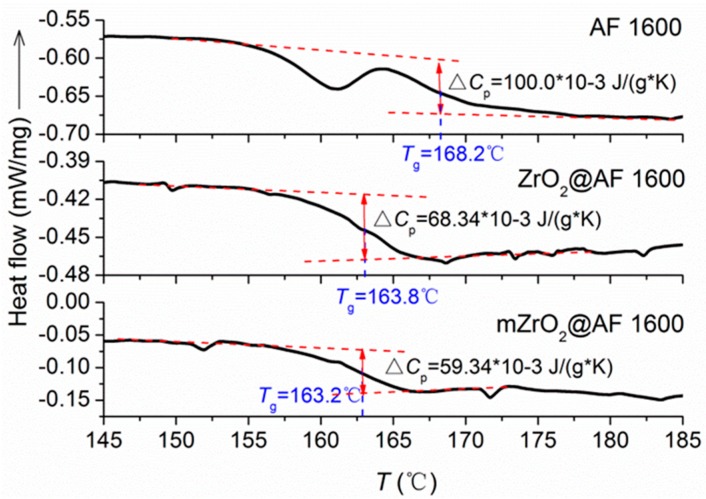
DSC heating curves of AF 1600, ZrO_2_@AF 1600 and mZrO_2_@AF 1600 samples (Δ*C*_p_: the specific heat capacity).

Meanwhile, mZrO_2_ nanoparticles have a good compatibility with AF 1600 substrate. In the temperature transformation diagrams (Figure 3), crude AF 1600 exhibits two endothermic peaks, but only one peak after hybridization. Both glass transition temperature (*T*_g_) and the specific heat capacity (*ΔC*_p_) fall, along with the introduction of ZrO_2_ and mZrO_2_ nanoparticles, respectively. Obviously, ZrO_2_ particles with good thermal conductivity and high specific surface area can conduct heat rapidly and inhibit the intertwining of polymer molecular chains effectively as a result of the “easier” phase transition. Additionally, these surface-fluorinated mZrO_2_ particles further decrease *T*_g_ and *ΔC*_p_ of nanocomposites, which only attributes to their better compatibility with AF 1600 than ZrO_2_’s. As previously mentioned, good matrix compatibility can lower the interfacial difference between nanofiller and framework material to make the nanocomposites more homogeneous. It will surely help tuning, even optimizing, some key functionalities for electrowetting intensification.

In the EWOD device, the dielectric layer does perform as a capacitor blocking electron and hold large electrostatic energy. Once an electric potential is applied between the top and bottom electrode, an electric double layer will appear spontaneously both at the solid–liquid interface and the solid–solid interface to cause the originally hydrophobic layer surface to be hydrophilic. Hereinto, the ability of charge accumulation, or in a sense, the surface wettability variation, depends entirely on dielectric constant (*ε*) of the resulting insulating layer. In general, *ε* is obtained by calculation according to the following equation:

(1) $$C=\varepsilon \varepsilon_{0}/t$$

where *C* is the capacitance per unit area, *t* is the thickness of the capacitor (i.e., dielectric layer between droplet and a solid electrode), *ε*_0_ is the permittivity of free space which is approximately 8.85 × 10^−12^ F m^−1^.

**Figure 4:**
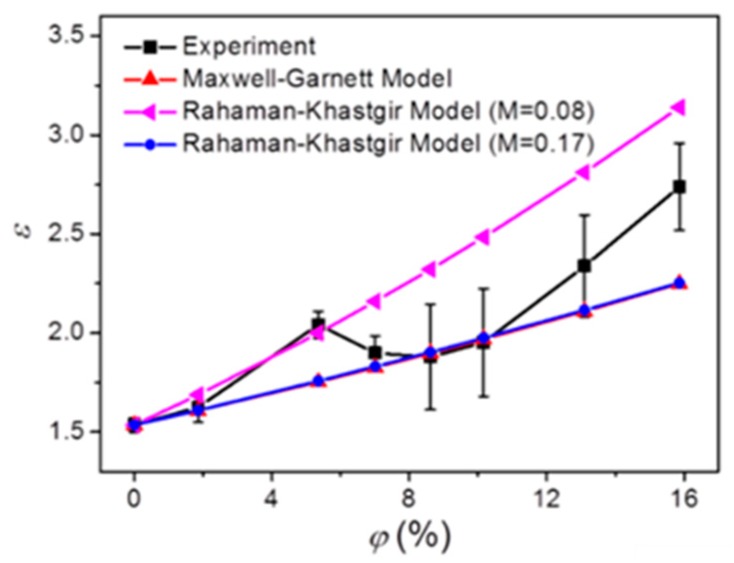
Experimental and theoretical dielectric constants (*ε*) of mZrO_2_@AF 1600 nanocomposite films under different mZrO_2_ volume fractions (*φ*).

Here, the obtained initial value of pure AF 1600 is only 1.54 that agrees with few measured data [45], but not with the general value (1.93). Just as shown in Figure 4, we investigated the relationship between *ε* value of mZrO_2_@AF 1600 nanocomposite with mZrO_2_ concentration in detail.

The *ε* equaling to 2.02 at low volume fraction of mZrO_2_ (5.35 vol %, i.e., 15 wt %) is higher than the theoretical value (1.75) calculated by Maxwell–Garnett model [46]:

(2) $$\varepsilon \;=\;\varepsilon_{m}\;\frac{2\varepsilon_{m}+\varepsilon_{f}+2\varphi (\varepsilon_{f}-\varepsilon_{m})}{2\varepsilon_{m}+\varepsilon_{f}-\varphi (\varepsilon_{f}-\varepsilon_{m})}$$

where *φ* represents the volume fraction of mZrO_2_ in AF 1600 matrix, *ε*, *ε*_m_ and *ε*_f_ denote the dielectric constant of the mZrO_2_@AF 1600 nanocomposite, AF 1600 matrix and mZrO_2_ filler, respectively. The inconsistence between the theoretical and experimental values of *ε* maybe attribute to the neglecting of interfacial polarization in Maxwell–Garnett model.

On the contrary, the Rahaman–Khastgir model involving the effect of interfacial polarization fits well with experimental data [47]:

(3) $$\log\varepsilon =\log\varepsilon_{f}+\frac{\log\varepsilon_{m}-\log\varepsilon_{f}}{e^{\frac{\varphi }{M*\log\;F}}}$$

where *F* is the measurement frequency (i.e., 1 kHz), and *M* is the morphological factor/fitting parameter, depending on shape, size, dispersion, distribution, and permittivity of particulate inclusion and host medium. It means that the improvement of dielectric constant at low mZrO_2_ content attributes to the interfacial polarization.

**Figure 5:**
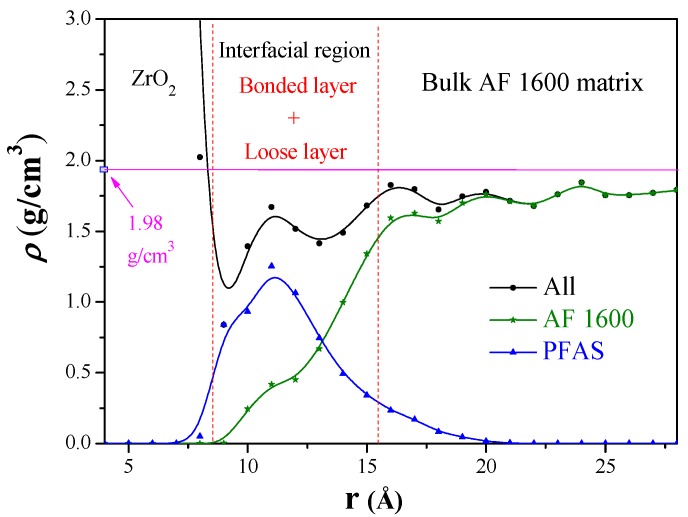
Density distribution around the center of ZrO_2_ in the mZrO_2_@AF 1600 nanocomposite. The content of mZrO_2_ is 5 wt %, and the radius of mZrO_2_ is approximately 8 nm.

For further understanding the interfacial effect of the composite, we adopted the molecular dynamics simulations with explicit atom model. Unlike the Tanaka’s multi-core model [48] of polymer nanocomposite (including the bonded layer, bound layer and loose layer), there is no bound layer in the mZrO_2_@AF 1600 nanocomposite. The density of the matrix of composite is lower than that of pure AF 1600 (1.98 g cm^−3^ based on the MD’s result), as shown in Figure 5. On the other hand, since the PFAS molecules do not cover the ZrO_2_ particle, the bonded layer and the loose layer are overlapped. The interfacial region comprising of a bonded/loose layer possesses an ultralow density. Low density indicates high free volume where the orientation of dipole moment can be rearranged easily under the electric field. In other words, the orientational polarization in the interfacial region is greater than that in the matrix. Therefore, the poor packing would account for faster segmental and chain dynamics, i.e., the improvement of the dielectric constant [49].

**Figure 6:**
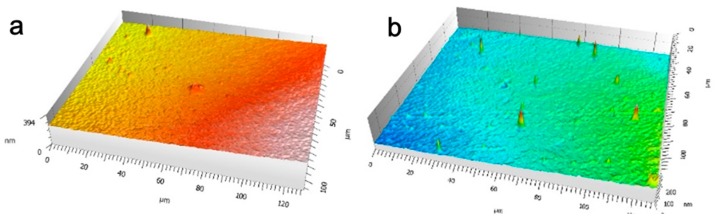
3D surface topography of AF 1600 (**a**) and mZrO_2_@AF 1600 with 5.35 vol % mZrO_2_ (**b**) monolayer film under 50-fold objective.

Given the above analyses, 5.35 vol % mZrO_2_@AF 1600 nanocomposite was chosen as the unique target of dielectric material to fabricate different dielectric layer structures in follow-up research. The surface morphology of dielectric film mainly decides the response rate and the reversibility of a droplet on the film under the applied electric field. Addition of inorganic fillers inevitably increases the surface roughness of organic polymer film. In Figure 6, the resulting mZrO_2_@AF 1600 film is almost as smooth as the AF 1600 one. Actually, its surface roughness (*R*a = 3.49 nm) is only slightly higher than the one of the latter (*R*a = 3.16 nm). Low surface roughness is attributed to low concentration and surface modification of inorganic fillers.

**Figure 7:**
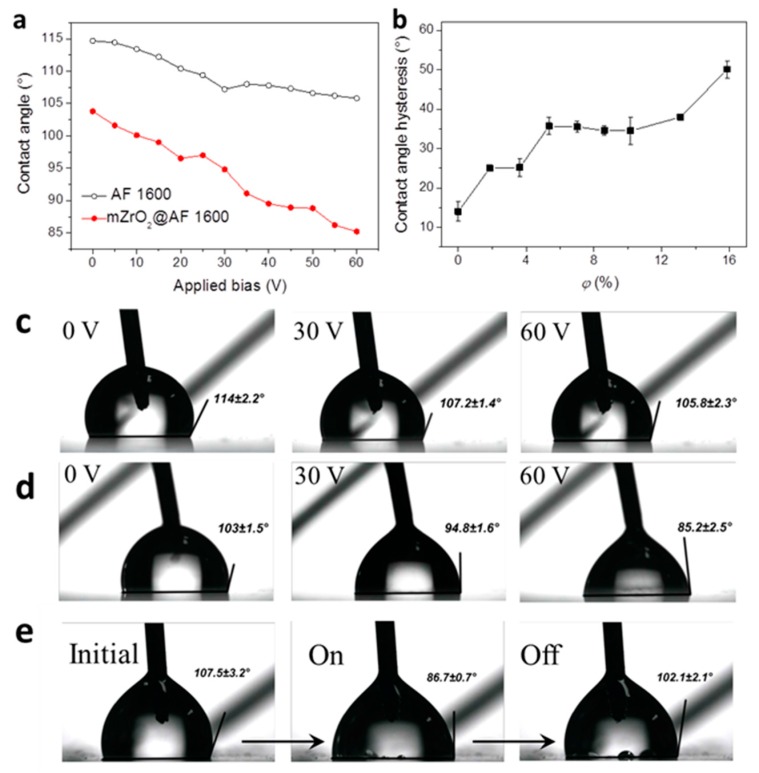
(**a**) Contact angles of the two films under applied DC electric field; (**b**) Contact angle hysteresis of the nanocomposite film with different mZrO_2_ concentrations; (**c**) Contact angle images of the monolayer AF 1600 and (**d**) mZrO_2_@AF 1600 film at 0, 30 and 60 V, respectively; (**e**) Electrowetting reversibility of the sandwich structure.

After analyzing the dielectric constant and the surface roughness which are the crucial factors for the wettability, the contact angles are investigated. Figure 7a,c,d shows that, although the initial contact angle of the single mZrO_2_@AF 1600 layer (103° ± 1.5°) is lower than that of the AF 1600 layer (114° ± 2.2°), its variation range of contact angles under applied DC electric field (17.8°) exceeds two times the AF 1600 one (8.2°). Very clearly, the larger contact angle modulation is based on higher dielectric constant of mZrO_2_@AF 1600 nanocomposite according to the Young–Lippmann equation:

(4) $$\cos\theta_{v}=\cos\theta_{0}+\frac{1}{2\gamma }\varepsilon \;\varepsilon_{0}\;U^{2}$$

where *θ*_0_ is the initial contact angle of droplet, *θ*_v_ is the contact angle under applied electric potential (*U*), *γ* is the surface tension between two immiscible fluids. However, its mildly wavy surface may bring about the drop of initial contact angle (see Figure 7a) and the rise of contact angle hysteresis (see Figure 7b). As the mZrO_2_ content rises, the dielectric layer becomes rougher to increase contact angle hysteresis against EWOD. Typically, the retreat ability and reproducibility of surface droplet will be weakened.

It is also inevitable that some mZrO_2_ nanoparticles in the AF 1600 matrix next to the electrode substrate will be partially polarized by ionization. Therefore, we further developed a three-layer sandwich structure with a top and bottom pure AF 1600 layer, and a mZrO_2_@AF 1600 interlayer, to make up for the above defects. As presented in Figure 7e, if the voltage is on state, the difference of surface contact angle of the sandwich film (20.8°) is higher than that of the single AF 1600 layer (8.2°). Once the voltage changes to be off state, the difference between the initial contact angle and the recovered contact angle of the former is only 5.4°. Meanwhile, its driving-recovery process can work very rapidly and repeatedly. These results indicate that the sandwich structure containing mZrO_2_@AF 1600 layer possesses better electrowetting reversibility and wider wettability modulation. Apparently, the enhanced electrowetting reversibility is attributed to the reduced surface roughness based on the top AF 1600 layer, and the widened wettability variation is attributed to the increased dielectric constant based on the mZrO_2_@AF 1600 nanocomposite interlayer.

**Figure 8:**
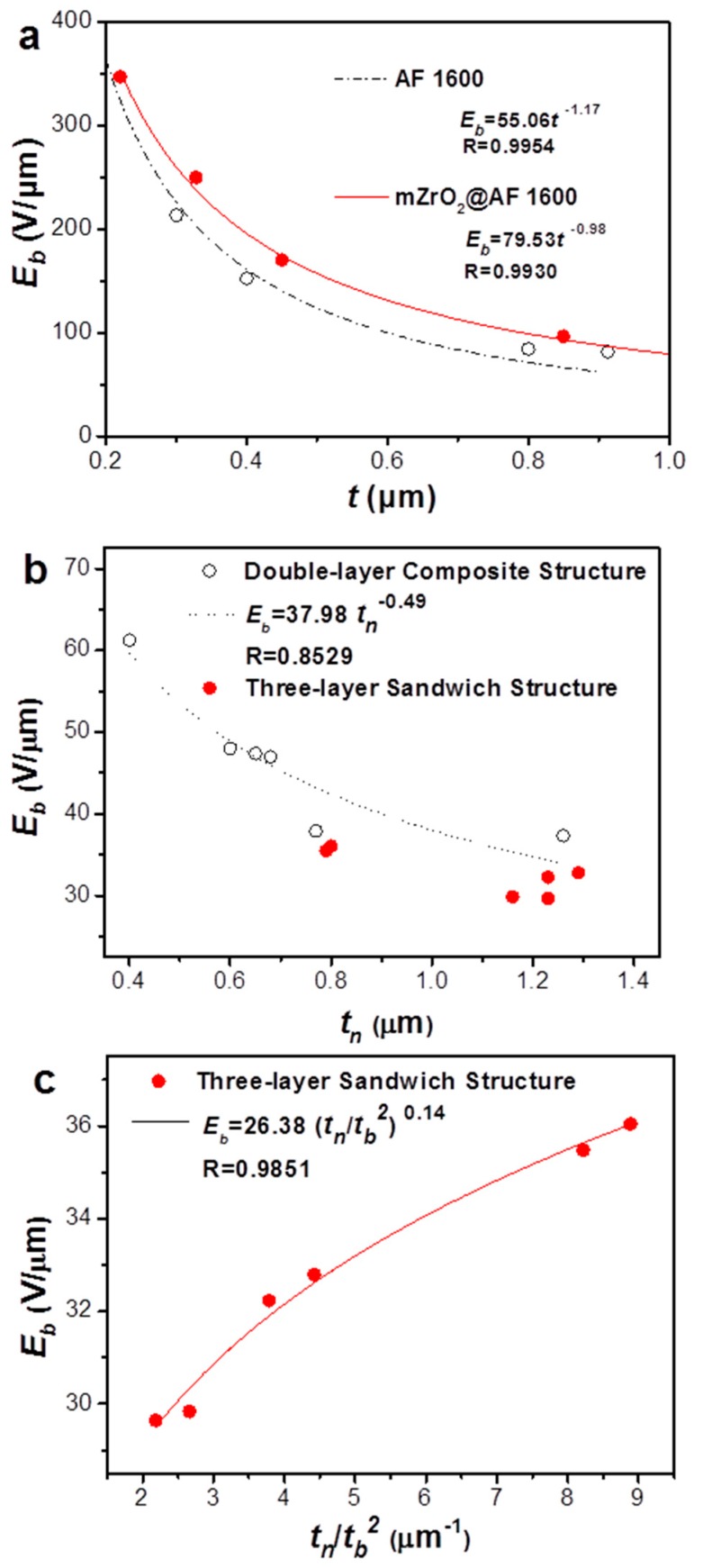
Mathematical relationship between thickness and breakdown field strength (*E*_b_) of (**a**) monolayer AF 1600 and mZrO_2_@AF 1600 film; (**b**) double-layer and (**c**) three-layer, respectively. Note that, *t* is total thickness, *t*_n_ is thicknesses of the mZrO_2_@AF 1600 layer (mZrO_2_ content: 5.35 vol %) and *t*_b_ is the thicknesses of the bottom AF 1600 layer.

Besides surface wettability, both breakdown voltage and corresponding breakdown field strength (*E*_b_) are considered as important evaluation indexes on the dielectric layer in EWOD. Based on the Young–Lippmann equation (Equation (4)), the *E*_b_ has a greater effect than *ε* on the contact angle modulation. Besides the nature of material, the thickness of the film has obvious influence on the breakdown field strength. According to the Forlani–Minnaja theory [50], the empirical relationship between *E*_b_ and thickness (t) appears as:

(5) $$E_{b}\propto t^{-k}$$

where *k* is determined by the cooperation of lattice energy state, electron energy state and electron–lattice interaction. Therefore, we fabricated a series of monolayer mZrO_2_@AF 1600 and AF 1600 films with thickness varying from 0.12 to 1.70 μm for *E*_b_ analysis. As shown in Figure 8a, the mZrO_2_@AF 1600 layer exhibits higher *E*_b_ value than AF 1600’s at the same thickness, similar to Dawber’s [51] and Huang’s [52] works on other amorphous solids. Obviously, it behaves with a higher proposed coefficient (79.53) and much closer to an ideal reciprocal relationship to *t* (*k* = −0.98). Here, the higher dielectric constant of the uniform nanocomposites originated from lack of ZrO_2_ aggregation plays an important role at low turning concentration.

What is the function of the bottom AF 1600 layer? Besides the surface roughness, we also measured and analyzed the breakdown performances of double-layer composite structure and three-layer sandwich structure presented in Figure 8b,c. Obviously, the double-layer structure approximately obeys the basic principle of *E*_b_ that decreases followed by increasing layer thickness (see Figure 8a), but the sandwich structure does not, as shown in Figure 8b. In Figure 8c, the best fitting curves reveal that, *E*_b_ of the sandwich structure mainly depends on the determined thickness of both the mZrO_2_@AF 1600 interlayer and the bottom AF 1600 layer, and may be not affected by the top AF 1600 layer. Particularly, according to the fitting equation, high *E*_b_ is very possible to be obtained by rising *t*_n_ and falling *t*_b_ simultaneously. Additionally, the higher volume fraction of the mZrO_2_@AF 1600 interlayer (*φ*_n_) and the resulting higher dielectric index (*ε*) also does. On the contrary, the opposite laws of layer thickness with *ε* and *E*_b_ (see Figure 8b,c) do not allow the double-layer composite structure to keep both *ε* and *E*_b_ gentle at a relatively high level for electrowetting intensification. Here, it is found that the bottom AF 1600 layer is of decisive importance in the three-layer sandwich structure. Just as mentioned previously, inorganic metal oxide with high polarity is very easily ionically polarized as a result of dielectric lose efficiency, but the bottom AF 1600 layer prevents its occurrence.

## 4. Conclusions

In conclusion, in order to optimize the dielectric performances of the insulating layer in EWOD, we combined theoretical models, molecular dynamics simulations, and experimental methods. In the experiments, we fabricated a polymer nanocomposite that combines the advantages of inorganic materials, fluoropolymer, and organic/inorganic composite structure. Here, the interfacial effect plays an important and conclusive role in outstanding electrowetting intensification. Firstly, surface-fluorinated modification of ZrO_2_ nanoparticles improves the compatibility of nanoparticles and the AF 1600 matrix to increase the dielectric constant of mZrO_2_@AF 1600 nanocomposite (2.02, about 31% higher than pure AF 1600’s) at low content, followed by double contact angle modulation and higher breakdown field strength. The small addition of mZrO_2_ improves the dielectric constant of the nanocomposite, and the experimental value is larger than the theoretical value calculated by the Maxwell–Garnett model, but fits well with the Rahaman–Khastgir model. The molecular dynamics simulations with the explicit model further verifies the interfacial effect. 

Its only weakness is that the nanoparticle hybridization surely generates worse contact angle hysteresis. In the sandwich structure, both the top and bottom AF 1600 layer may weaken the bumpy medial mZrO_2_@AF 1600 layer for better electrowetting reversibility and wider wettability modulation. At the same time, the bottom layer isolated those ZrO_2_ nanoparticles from the electrode against ionic polarization. Marvelously, this structure can change the relationship between volume fraction of mZrO_2_@AF 1600 layer and *E*_b_, which will make more potential spaces for overall enhancements on both dielectric and breakdown performances. Multiscale interface effects based on the surface modification of nanoparticles and design of sandwich structure are revealed. It is anticipated to provide a facile, effective and practical strategy for enhancing the electrowetting performances of EWOD.
